# Fsh Controls Gene Expression in Fish both Independently of and through Steroid Mediation

**DOI:** 10.1371/journal.pone.0076684

**Published:** 2013-10-23

**Authors:** Elisabeth Sambroni, Jean-Jacques Lareyre, Florence Le Gac

**Affiliations:** INRA, UR1037 LPGP, Testicular Physiology and Puberty, SFR BIOSIT, Biogenouest, Campus de Beaulieu, Rennes, France; University Hospital of Münster, Germany

## Abstract

The mechanisms and the mediators relaying Fsh action on testicular functions are poorly understood. Unlike in mammals, in fish both gonadotropins (Fsh and Lh) are able to efficiently stimulate steroidogenesis, likely through a direct interaction with their cognate receptors present on the Leydig cells. In this context, it is crucial to understand if Fsh effects are mediated through the production of steroids. To address this issue we performed transcriptome studies after *in vitro* incubations of rainbow trout testis explants in the presence of Fsh alone or in combination with trilostane, an inhibitor of Δ4- steroidogenesis.

Trilostane significantly reduced or suppressed the response of many genes to Fsh (like *wisp1*, testis *gapdhs*, *cldn11*, *inha*, *vt1* or *dmrt1*) showing that, in fish, important aspects of Fsh action follow indirect pathways and require the production of Δ4-steroids. What is more, most of the genes regulated by Fsh through steroid mediation were similarly regulated by Lh (and/or androgens). In contrast, the response to Fsh of other genes was not suppressed in the presence of trilostane. These latter included genes encoding for anti-mullerian hormone, midkine a (pleiotrophin related), angiopoietine-related protein, cyclins E1 and G1, hepatocyte growth factor activator, insulin-like growth factor 1b/3. A majority of those genes were preferentially regulated by Fsh, when compared to Lh, suggesting that specific regulatory effects of Fsh did not depend on steroid production. Finally, antagonistic effects between Fsh and steroids were found, in particular for genes encoding key factors of steroidogenesis (*star*, *hsd3b1*, *cyp11b2-2*) or for genes of the Igf system (*igf1b/3*). Our study provides the first clear evidence that, in fish, Fsh exerts Δ4-steroid-independent regulatory functions on many genes which are highly relevant for the onset of spermatogenesis.

## Introduction

In vertebrates, reproductive function is under the control of multiple factors acting in a cascade of regulations known as the brain-pituitary-gonadal (BPG) axis.

The male gonad is separated into two compartments, each with specific functions: the tubular compartment where spermatogenesis takes place to produce spermatozoa and the interstitial compartment which produces most of the steroids. Spermatogenesis is under the dual control of gonadotropins and sexual steroids. In mammals, each of the two gonadotropins, FSH and LH, have well targeted actions on the testis due to the exclusive presence of FSH receptors in the seminiferous epithelium, mostly on Sertoli cells and of LH receptors in the interstitial tissue. Despite rare reports on FSH receptors being detected in the germ cell line [1], the general consensus is that germ cell development is supported indirectly by gonadotropins. FSH stimulates the Sertoli cells to produce extracellular matrix proteins, growth factors and cytokines which in turn regulate germ cell proliferation and differentiation. LH stimulates the production of androgens by the Leydig cells. Androgens are required for normal development of spermatogenesis as illustrated by the impaired spermatogenesis in mutant mice lacking the nuclear androgen receptor either specifically in Sertoli cells (SCARKO) or in all testicular cell types expressing the nuclear AR (ARKO) [2], [3].

This scheme is probably more complex since, at least in rodents, FSH action may involve or require the steroid pathway. In *hpg* mice that are devoid of gonadotropin production, FSH supplementation has been shown to induce Leydig cell function, probably indirectly through Sertoli cell stimulation [4], [5]. Moreover, the development of round spermatids induced by FSH requires androgen action since the FSH effect is suppressed in *hpg*.SCARKO or *hpg*.ARKO mice [6].

The situation seems quite different in teleost fish, since in these species it has been established that both gonadotropins efficiently up-regulate steroidogenesis in testis and ovary, although some gonadal maturation stages can be more sensitive to one or the other gonadotropin [7]–[9]. This stimulating effect of Fsh and Lh is achieved through the up-regulation of genes encoding key players of steroid synthesis [10]–[12]. This particular feature can be explained by the expression of Lh receptors and Fsh receptors in the same interstitial cell type - probably Leydig cells - a situation described in an increasing number of primitive or evolutionarily advanced teleostean fish species like eel [13], African catfish [14], zebrafish [12], honeycomb grouper [15] and Senegalese sole [16]. In rainbow trout, this scheme ought to explain the potency of Fsh to stimulate steroidogenesis since we showed that Fsh was not able to activate the Lh receptor efficiently [17].

Both Fsh and androgens are involved in the onset of spermatogenesis in fish. In Japanese eel, recombinant eel Fsh or 11-ketotestosterone (11KT) induced all steps of spermatogenesis from immature testicular explants cultured *in vitro* [18]. Moreover, the Fsh-induced spermatogenesis was inhibited by trilostane. The authors concluded that in this species the main role of Fsh in spermatogenesis would be to induce the production of 11KT, which in turn would regulate Sertoli cell function and, indirectly, germ cell proliferation/differentiation [13]. However in sea bass, Fsh but not Lh, was able to trigger spermatogenesis *in vivo* [19]. In salmonids, Fsh is the only circulating gonadotropin during the early stages of the reproductive cycle when A-spermatogonia actively proliferate and commit into differentiation [20]. Furthermore, Fsh induces the proliferation of spermatogonia in mixed cultures of trout somatic and germ cells [21]. In contrast, testosterone or 11KT had no effect on spermatogonia proliferation [21]. Thus, part of Fsh action is likely mediated by steroids but these latter may not fulfill all Fsh functions required to initiate spermatogenesis. In teleosts little is known about Fsh actions that would be independent of steroids. We recently demonstrated that *in vitro* Fsh treatment greatly modified the testicular transcriptome in trout and that this effect significantly differs from the effect of Lh [10]. The present work aimed at distinguishing the steroid independent actions of Fsh on testicular gene expression from those mediated by the steroids. Using a large scale transcriptomic analysis, we clearly demonstrate that Fsh acts both independently of and through the Δ4-steroid production. In addition, our data suggest that the specific regulatory effects of Fsh (in comparison to Lh), were mainly independent of steroid production. Finally, we find that Fsh and steroids may also have antagonistic regulatory effects, underlining the complex coordinated regulation of spermatogenesis by the different reproductive hormones.

## Materials and Methods

### Animals and *in vitro* organotypic culture

#### Ethics statement

Animals were bred and treated according to the guidelines for the use and care of laboratory animals and in compliance with French and European regulations on animal welfare. This project was approved by the local animal care and ethics committee of INRA under agreement n° B0009. The personnel were trained and qualified for animal experimentations.

#### Procedure

An all-male population of rainbow trout (*Oncorhynchus mykiss*) was obtained from the INRA experimental fish farm (PEIMA, Drennec, France) and kept in the laboratory facilities at 12°C under natural photoperiod until experimentation. Fish were deeply anesthetized in 1‰ 2-phenoxyethanol then killed by a blow to the head. Testes were removed, weighed and kept on ice in synthetic L15 media as modified by Loir [22] until preparation for culture. According to the macroscopic aspect of the testes and to the calculated gonadosomatic index (GSI), only fish with GSI≤0.15% were kept. Gonads were collected and cut into 1 mm^3^ pieces using an automatic tissue chopper. All explants were pooled and mixed and about 10 testis fragments were randomly distributed (60–80 mg per well) onto Nunc polycarbonate membrane inserts in 24-well plates filled with 300 µL of culture medium supplemented with 2% Ultroser SF. Six replicate wells were used for each treatment. Incubation was performed for 96 h, at 12°C. Medium and hormones were replaced after 48 h of incubation. At the end of the incubation, tissues and culture media were centrifuged for 10 min at 200*g*. Tissues were frozen at −80°C in 1.2 mL of TRIzol® until RNA extraction. Culture media were frozen at −20°C until steroid radioimmunoassay.

Experiment 1 was carried out to evaluate the transcriptomic action of Fsh in the presence of trilostane, an inhibitor of the 3 beta-hydroxysteroid dehydrogenase. The pool of testis explants was issued from 58 fish (GSI mean = 0.089±0.037%). Explants were incubated in the absence or the presence of purified pituitary salmonid Fsh (500 ng/mL) alone or in combination with 10 µg/mL trilostane (C_20_H_27_NO_3_, CHEMOS GmbH, Germany). A pre-incubation with trilostane was carried out for 1 hour before adding Fsh. These samples were used for the large scale transcriptomic analysis on trout cDNA nylon membrane arrays.

The same conditions were used in Experiment 2 to evaluate the action of Fsh and androgens on the transcription of a few candidate genes selected from Experiment 1. The pool of testis explants originated from 26 fish (GSI mean = 0.104±0.028%). Explants were incubated in the absence or the presence of purified Fsh (500 ng/mL) and of 2 biologically active androgens: 11-ketotestosterone (11KT) and 17α-methyl testosterone (MT, 17α-methyl-4-androsten-3-one) at the concentration of 300 ng/mL (about 10^−6^ M). This concentration was close to the 11KT concentration measured in the culture medium after 48 h of incubation with Fsh in Experiment 1.

A few pieces of testis tissue were also fixed on the day of sampling in Bouin's solution for histological examination. Fixed gonads were dehydrated and embedded in paraffin, and 5 µm sections were cut and stained with Regaud-Haematoxylin-Orange G-Aniline blue. The maturity stage of the gonads was evaluated based on the presence and on the relative abundance of the most developed germ cells, according to a classification described previously [20]. In each well, about 50% of explants were in stage I–II of testis maturation. These two gonadal stages are characterized by the presence of a large majority of A-spermatogonia. The remaining explants corresponded to Stage III which is characterized by a large number of B-spermatogonia and the appearance of meiotic cells (spermatocytes and rare spermatids).

### Steroid measurement

To denature steroid binding proteins which may interfere with the steroid antibody, media were heated at 60°C for 20 min and centrifuged at 3000*g*, at 4°C for 15 min. Levels of 11KT were measured by specific radioimmunoassay (RIA) in culture media from Experiment 1 according to Fostier et al. [23]. Each sample was assayed in duplicate. The assay sensitivity was 80 pg/mL and the cross reactivity with testosterone or adrenosterone was 10%. The inter- and intra-assay coefficients of variation were 15% and 6%, respectively.

### cDNA nylon membrane array experiment

#### RNA extraction and cDNA target synthesis

Total RNA was extracted using TRIzol® reagent and further purified with the NucleoSpin® RNA II kit (Macherey Nagel). RNA concentrations were quantified using the NanoDrop ND-1000 (Thermo Scientific) and RNA quality was determined using the Bioanalyser 2100 (Agilent). For cDNA target labeling, 5 µg total RNA were reverse-transcribed for 2 h at 42°C in the presence of radiolabelled dNTP (30 µCi [alpha-^33^P] dCTP, 120 µMdCTP, 20 mM each dATP, dTTP, dGTP) using an oligo(dT) primer and 400 units of Superscript II reverse transcriptase (Invitrogen). RNA was degraded at 68°C for 30 min with l µL 10% SDS, l µL 0.5 M EDTA and 3 µL 3 M NaOH. The reaction was then equilibrated at room temperature for 15 minutes and neutralized by the addition of 10 µL 1 M Tris-HCI and 3 µL 2 N HCl.

#### Nylon membrane array hybridization and raw data production

cDNA arrays were generated by CRB GADIE (http://crb-gadie.inra.fr/) as previously described [24]. Prehybridization of the membranes was performed at 65°C for 4 h in 5X Denhardt's, 5X SSC and 0.5% SDS. Labelled cDNA targets were denatured at 95°C for 5 min and incubated with microarrays for 48 h at 65°C in the same buffer. The membranes were then washed three times for 1 h at 68°C in 0.1X SSC containing 0.2% SDS prior to a 48 hour exposure to phosphor-imaging plates. Plates were scanned using a FUJI BAS 500 and the BZscan software was used for signals acquisition [25]. Each membrane was also hybridized with a ^33^P-labelled oligonucleotide (5′-TAATACGACTCACTATAGGG-3′) that recognizes the vector part of every PCR product to quantify the amount of spotted cDNA.

#### Normalization procedure

Expression data were normalized as previously described [26]. Briefly, raw data were corrected for the amount of spotted cDNA by dividing the sample signal (Si) of each spot by the corresponding vector signal (Vi). To avoid the bias affecting relative gene expression levels, the corrected signal of each spot was further multiplied by the median vector signal of all arrays for this same spot ((Si/Vi)×medVi). Expression values were then log2-transformed and submitted to a quantile-quantile normalization using the AMEN software (http://sourceforge.net/projects/amen/) [27]. Raw data as well as a normalized expression file are available at the GeneOmnibus public data repository http://www.ncbi.nlm.nih.gov/geo/query/acc.cgi?acc=GSE46458.

#### Statistical and cluster analyses

Non-informative clones for which too small an amount of cDNA was spotted (oligonucleotide signal <3 times the background level in more than 20% of samples) were removed from the analysis. Clones were further filtered on their expression level (mean expression level ≥ to the median expression of all the experiment, at least in one experimental group). Finally, clones were filtered on a fold change ≥1.5 between control and Fsh-treated groups either in the absence or in the presence of trilostane.

Gonadotropin-responsive genes were then identified in the presence or not of trilostane, by comparing control groups to the Fsh-treated groups using the multi-class Limma statistical test with a false discovery rate (FDR) of 1% [28]. All differentially-expressed transcripts were then submitted to a hierarchical classification (Uncentered Pearson correlation measure).

#### Meta-analysis

Expression data obtained in a previous study [10] were used to investigate whether the 2 categories of Fsh-responsive genes (sensitive or insensitive to trilostane), were regulated by Lh. Samples in this dataset included explants of testis in early stages of spermatogenesis which were incubated for 4 days in the absence or in the presence of either Fsh or Lh (500 ng/mL). The data were normalized to avoid bias due to the different batches of nylon membranes as well as the 2 separate hybridization experiments. For each row (i.e. each gene) the expression signals of all arrays in one experiment were median-centered then normalized by the median expression of the second experiment. Only the clones demonstrating a statistically significant response to Fsh in both experiments were considered and submitted to a non-supervised hierarchical classification.

### Real-time quantitative PCR (qPCR) experiments

The qPCR technique was used either to confirm changes in expression for selected transcripts identified from the microarray analysis or to examine additional transcripts previously found as being differentially regulated by gonadotropins and/or of putative interest regarding testis functions (the list is given in Table 1). Two micrograms of total RNA were submitted to reverse-transcription (RT) using 1 µg random hexamers and 200 units of MMLV reverse transcriptase (Promega) for 75 min at 37°C in a final of volume of 25 µL. Real-time PCR assays were performed on the StepOne™ Real-Time PCR System (Applied Biosystems) using 4 µL of 1∶30 diluted RT products, 1 µL of mixed oligo primers (0.6 µM for both reverse and forward primers) and 5 µL of Fast SYBR® Green Master Mix (Applied Biosystems). The amplification program consisted of an initial denaturation at 95°C for 20 sec; 40 cycles of 95°C for 3 sec, then 60°C for 30 sec. A final progressive increase of temperature (0.5°C/sec) has been carried out from 65 to 90°C at the end of the amplification for melting curve analysis.

**Table 1:** Additional candidate genes studied using qPCR.

SwissProt/GeneBank accession number	Gene Symbol	Annotation/Description	Preferential gonadotropin response	Steroid mediation	Predicted cellular origin
-	*igf1b/igf3*	Insulin-like growth factor 1b	Up Fsh	No, antagonism	Somatic
Q3HWG4	*igfbp6*	Insulin-like growth factor binding protein 6	Up Fsh	Partially	-
Q9I8S6	*cyp11b2-2*	Cytochrome P450 11 beta 2	Up Fsh, Lh	No, antagonism	-
Q71MM8	*fshr*	Follicle-stimulating hormone receptor	Up Fsh, Lh	Yes	Somatic
NM_001165391	*ccnd1*	G1/S-specific cyclin-D1	Down Fsh	No	-
Q71MM9	*lhcgr*	Luteinizing hormone receptor	Up Fsh, Lh	Yes	Somatic
O95633	*fstl3*	Follistatin-related protein 3 precursor	Up Fsh	No, antagonism	
NP_001117674	*star*	Steroidogenic acute regulatory protein, mitochondrial precursor	Up Fsh, Lh	No, antagonism	Somatic

These genes were of interest since they were previously found to be differentially regulated by gonadotropins (Sambroni et al., 2013).

Cycle threshold (Ct) was automatically setup and relative expression levels were normalized using a reference gene, *rps15* (clone 1RT58B15_B_A08). This gene was chosen on the basis of its invariant expression in spermatogenesis microarray experiments [26]. Its expression level also enabled its measurement at the same RT template dilution as selected candidate genes. All RT samples were measured in duplicates. Statistical analyses were performed with the Statistica software environment using the non-parametric ANOVA of Kruskal-Wallis and the Mann & Whitney's U test if a statistical difference (p<0.05) was observed between groups in the ANOVA analysis.

Real-time PCR oligonucleotide primers were designed using the Primer3 software (http://frodo.wi.mit.edu/primer3/) and were verified with the oligoanalyser 3.1 web interface (http://eu.idtdna.com/analyzer/Applications/OligoAnalyzer/) to avoid self- and hetero-dimer formation as well as hairpin structures. Nucleotide sequences of the primers were also systematically matched (BLAST algorithm) against the SIGENAE trout contig collection (som.10 version) to avoid non-specific annealing to other transcripts. PCR amplification effectiveness was verified using serial dilutions of pooled RT products. All primer sequences are provided in Table S1.

## Results

To address the issue of steroid-mediated action of Fsh on testicular transcriptome, we carried out *in vitro* culture of testis explants incubated in the presence of Fsh alone or in combination with trilostane (Fsh+Tri), a known inhibitor of the 3 beta-hydroxysteroid dehydrogenase.

### Trilostane efficiently suppressed basal and Fsh-stimulated androgen production

**Figure 1:**
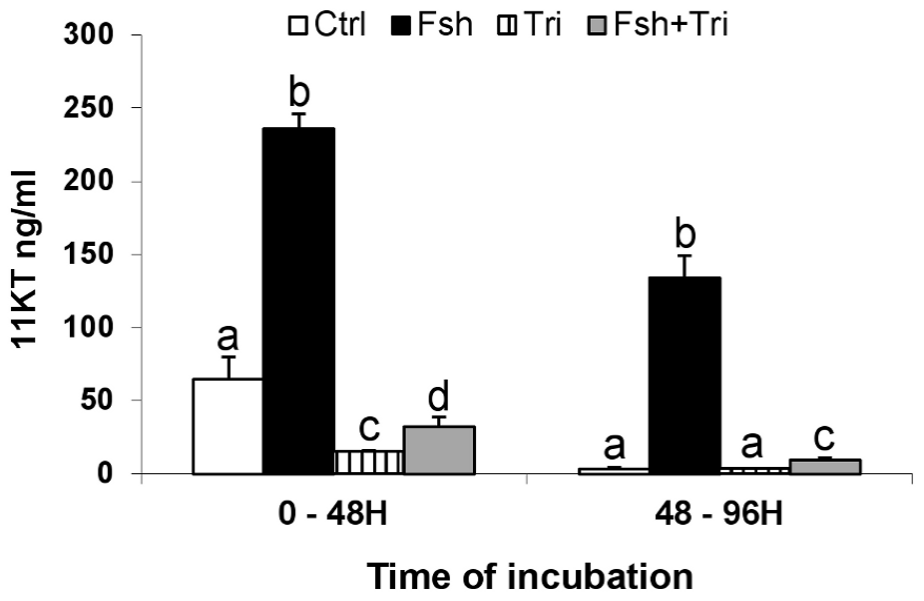
Evaluation of trilostane treatment efficiency. 11KT production in culture media after 48(500 ng/mL) alone or in combination with trilostane (10 µg/mL). Culture media were replaced after 48 h. Each bar represents the mean ± SD of 6 replicates. Different letters for each incubation duration indicate that treatments have significantly different effects as determined by non-parametric Mann & Whitney test (p<0.01).

To determine the effectiveness of trilostane (Tri) in inhibiting Δ4-steroid synthesis throughout the culture period, we measured 11KT levels in the culture media. As expected, Fsh alone induced a strong stimulation of the production of 11KT (about 5 fold) over the culture period (Fig. 1). After the first 48 hours of incubation, the presence of trilostane resulted in a reduction of both the basal and the Fsh-stimulated 11KT production (76% and 86% decrease, respectively). Over the following 48 hour period, the basal production of 11KT was drastically reduced so that the trilostane effect was no longer observed. The Fsh-stimulated production was maintained under control condition but was nearly suppressed (−93%) in trilostane treated explants. Consequently, testis tissues incubated in the presence of both Fsh and trilostane were exposed to much lower levels of androgens than testis tissues incubated in the presence of Fsh alone (9.4±1.4 versus 134.00±15.10 ng/mL at the end of the culture).

### Trilostane modified the Fsh regulation of testicular gene expression

**Figure 2:**
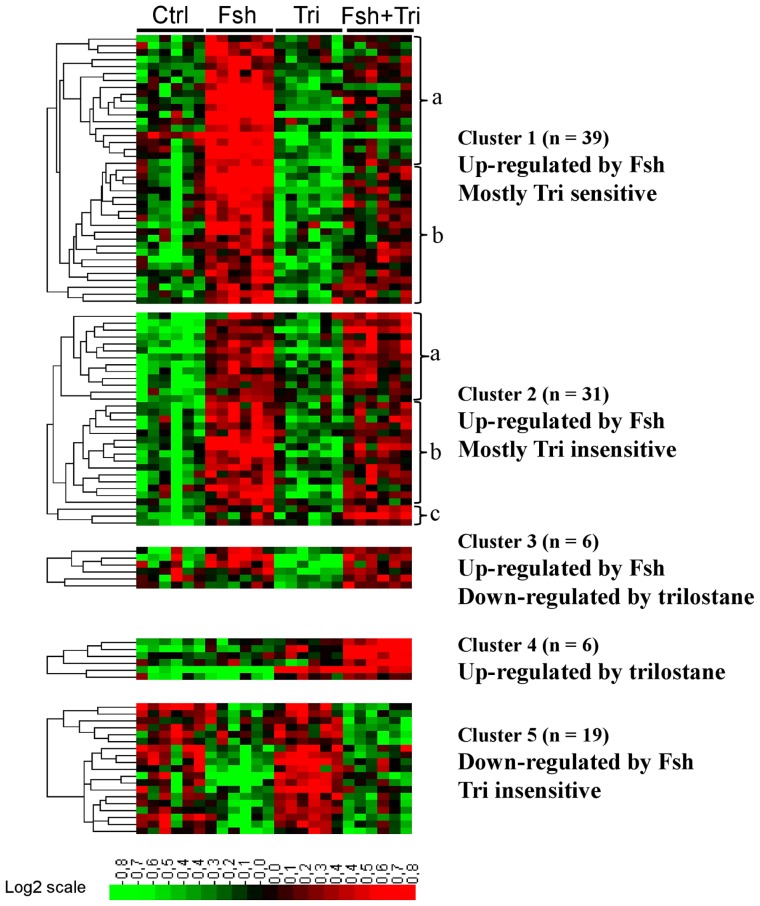
Expression of Fsh-responsive genes. Heatmap representation of the hierarchical classification of the 102 clones differentially regulated in trout testis after an *in vitro* 4-day incubation without any substance (Ctrl) or with Fsh alone at 500 ng/mL (Fsh), trilostane alone at 10 µg/mL (Tri) or with both Fsh and trilostane (Fsh+Tri). Trilostane was added 1 h before Fsh addition in the medium. Media and hormones were renewed after 2 days. Clones segregated into 5 main groups, corresponding to genes which were up- or down-regulated by Fsh and sensitive or not to trilostane. Normalized expression values are shown according to the scale bar. Each line represents a clone and each column is a sample.

The variations of the transcriptome were analyzed at a large scale using trout cDNA microarrays. The effects of Fsh on testicular gene expression were studied after 4 days of incubation because we previously showed that Fsh and Lh modified testicular transcriptome more efficiently after a 4-day treatment compared to shorter durations. The microarray data were analyzed with AMEN software. After a double filtration on expression level and fold change, followed by a Limma analysis (FDR 1%; see M&M), 102 clones corresponding to 96 non redundant (NR) genes were found significantly differentially expressed between control and Fsh-treated conditions or between trilostane and Fsh+trilostane groups. All the information on annotation together with response to Fsh, trilostane and Lh for these 102 clones is provided in the searchable file S1. (Note that the responsiveness to Lh was determined in a previous study [10]). The hierarchical classification of the genes allowed the segregation of 5 main clusters of transcripts with correlated variations along the samples (Fig. 2). Overall, the great majority (74 out of 102) of the differentially-expressed transcripts were found to be up-regulated by Fsh.

#### Fsh action mediated by steroid production

**Figure 3:**
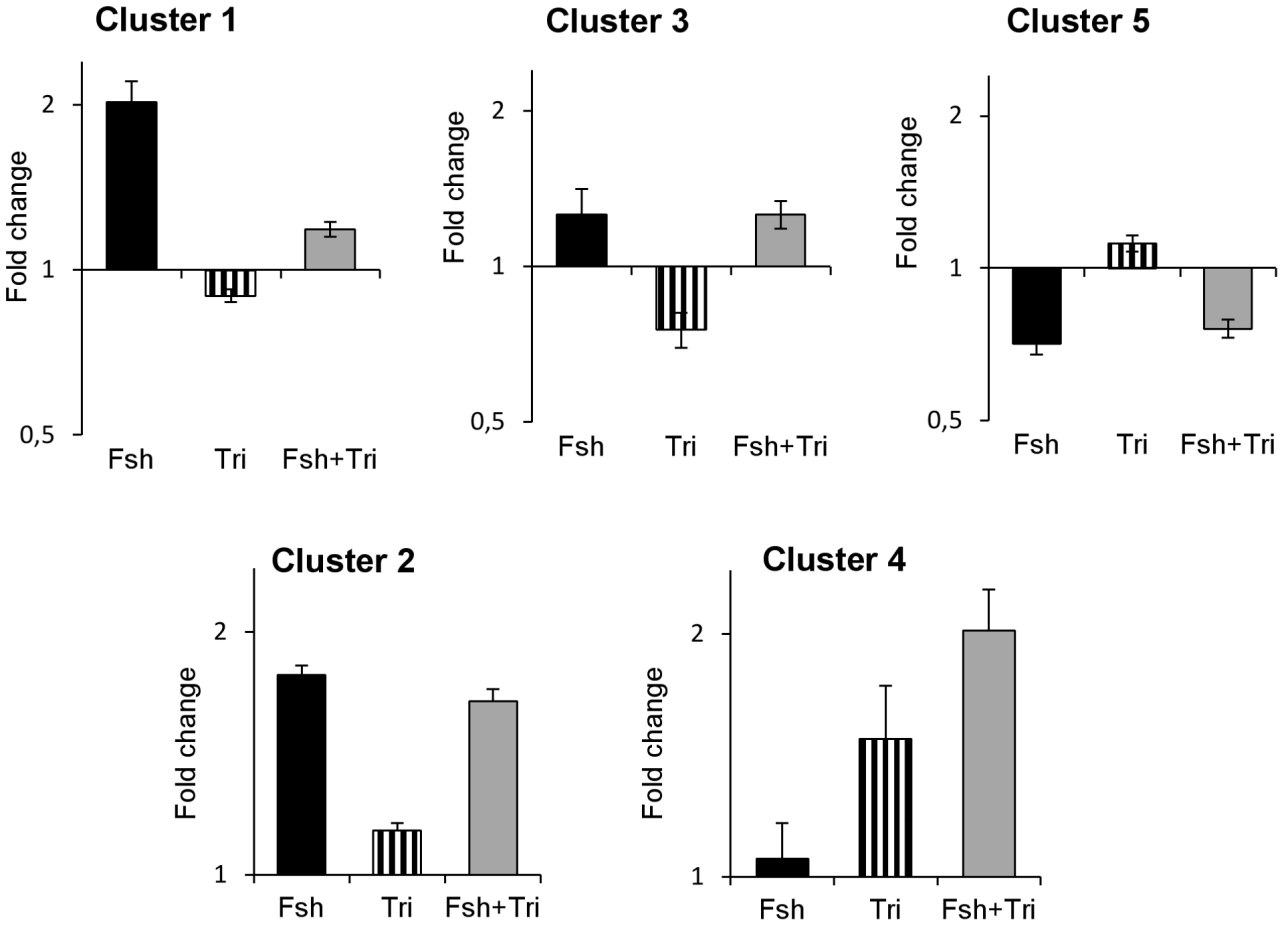
Representation of the mean fold-change in each cluster. Fold changes are relative to the control group. All the 102 clones included were individually found significantly affected by Fsh. Bars represent mean ± SEM. Y-axis is log-2 scaled and value 1 represents the control level. For convenience, note that the clusters are not shown in numerically ascending order.

**Figure 4:**
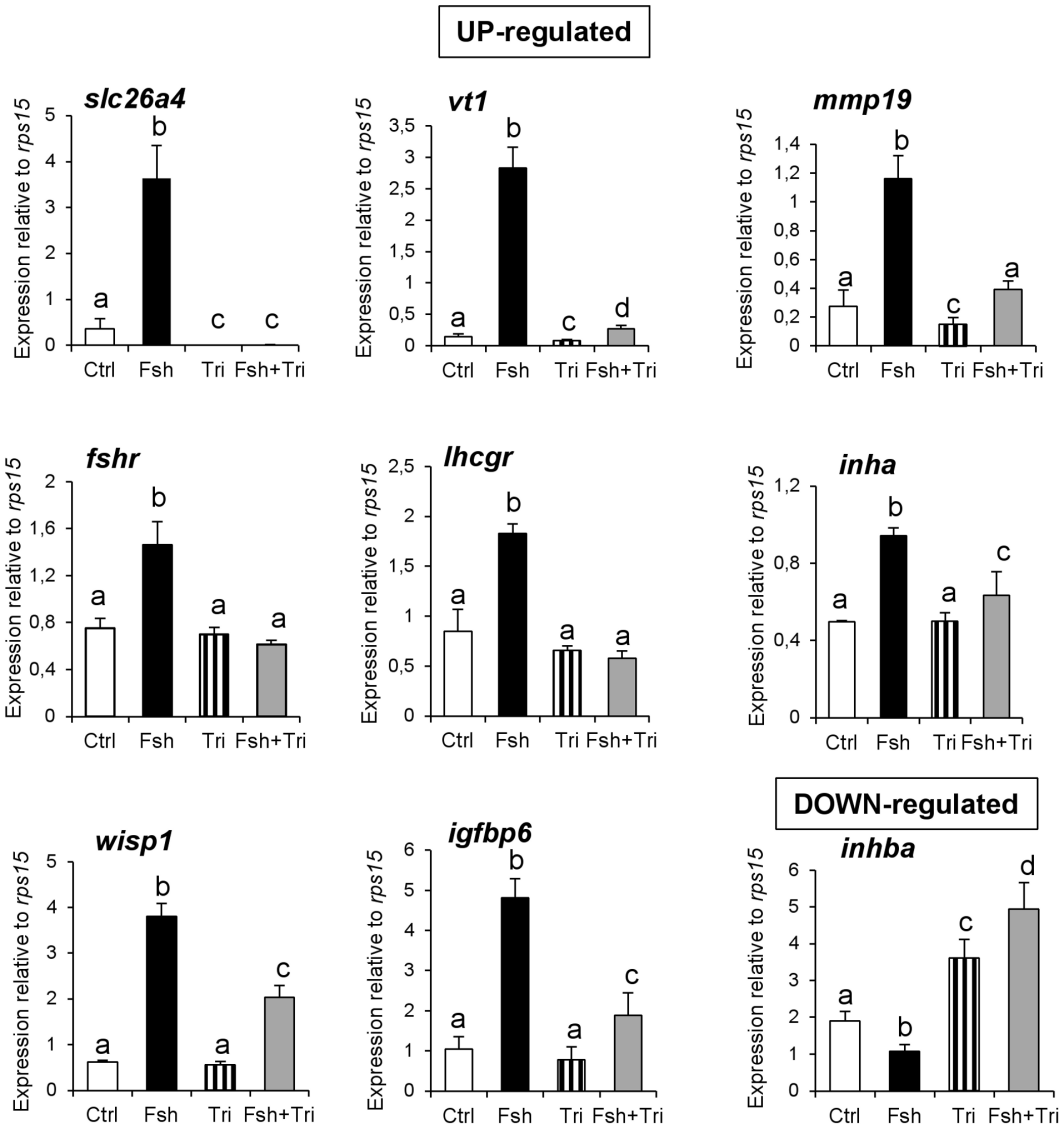
Steroid-mediated action of Fsh on the steady-state level of mRNA transcripts measured by qPCR. *slc26a4*, *vt1*, *mmp19* and *wisp1* and *inha* genes segregate in Cluster 1. The *inhba* gene belongs to Cluster 4. Three additional transcripts (*fshr*, *lhcgr* and *igfbp6*) previously demonstrated as up-regulated by gonadotropins are found to behave like genes in Cluster 1. Note that Fsh regulations of *inha*, *wisp1* and *igfbp6* are only partially lost in the presence of Tri. Bars represent mean ± SD of 5 to 6 replicates. Expression data were normalized to the reference gene *rps15*. Different letters indicate that treatments are significantly different as determined by non-parametric Mann & Whitney test.

In Cluster 1, 39 genes were up-regulated by Fsh and this up-regulation was greatly inhibited in the presence of trilostane (Fig. 3). This implies that the responsiveness of these genes to Fsh requires the production of steroids. Cluster 1 could be subdivided in 2 groups of genes: those for which the response to Fsh was suppressed by trilostane (Fig. 2, Cluster 1a) as for *nr2f2*, *gapdhs*, *slc26a4*, *smtn*, *ndpkz3*, *canx*, *fblim1*, *dmd* or *cebpd1* and those for which the response to Fsh was strongly reduced but not totally inhibited (Fig. 2, Cluster 1b) as for *dmrt1*, *cldn11*, *etnk1*, *cth*, *plg*, *sptbn1*, *vt1*, *timp2* and *mmp19* (Table 2). For some representative genes of Cluster 1, we confirmed by qPCR that the presence of trilostane led to a complete or drastic loss of the response to Fsh, supporting the hypothesis that Fsh indirectly regulated their expression through the production of steroids (Fig. 4). The up-regulation of three additional transcripts *fshr*, *lhcgr* and *igfbp6* was also found to be mediated by steroids.

**Table 2:** Representative transcripts up-regulated by Fsh and sensitive to trilostane treatment.

Gene Symbol	Gene description	Lh regulation^1^	Testicular expression profile^2^	*In vivo* androgen response^3^
**Fsh-response abolished by trilostane treatment**				
-	14 kDa apolipoprotein	Yes	Somatic	-
*cebpd1*	CCAAT/enhancer-binding protein delta (C/EBP delta)	Yes	Somatic	-
*nr2f2*	COUP transcription factor 2 (COUP-TF2)	No	-	-
*canx*	Calnexin precursor	-	Somatic Up stage 8	-
*-*	Cytokine-like nuclear factor n-pac	-	-	-
*cyp46a1*	Cytochrome P450 46A1	No	Somatic	Up at day 7
*dmd*	Dystrophin	Yes	Meiotic Up stage 8	-
*fdps*	Farnesyl pyrophosphate synthetase (FPP synthetase)	-	-	-
*fblim1*	Filamin-binding LIM protein 1 (FBLP-1)	-	-	-
*gapdhs*	Glyceraldehyde-3-phosphate dehydrogenase, testis-specific	-	-	-
*it*	Isotocin-I	Yes	Somatic Up stage 8	-
*-*	Similar to vertebrate acyl-CoA thioesterase 11	-	A-spermatogonia	-
*slc26a4*	Similar to vertebrate solute carrier family 26	Yes	Somatic Up stage 8	Up at day 7
*ndkpz3*	Nucleoside diphosphate kinase-Z3	Yes		-
*-*	Putative uncharacterized protein (Fragment)	Yes	Somatic Up stage 8	-
*smtn*	Smoothelin-b (Fragment)	No	Somatic	Up at day 14
*-*	Similar to vertebrate protein tyrosine phosphatase, receptor type, F	Yes	-	-
*tpd52l1*	Tumor protein D53 homolog	Yes	-	-
*wisp1*	WNT1-inducible-signaling pathway protein 1 precursor	No	Somatic Up stage 8	-
**Fsh-response significantly reduced by trilostane treatment**				
*ctl1*	Cathepsin L	Yes	Somatic	-
*cebpd2*	CCAAT/enhancer binding protein delta2	Yes	Somatic	-
*lrrc8*	Leucine-rich repeat-containing protein 8C	Yes	-	-
*cldn11*	Claudin 11a	-	Somatic Up stage 8	Up at day 7
*cth*	Cystathionase (cystathionine gamma-lyase)	Yes	Somatic Up stage 8	Up at day 7
*cyp2m1*	Cytochrome P450 2M1	Yes	Somatic	Up at day 7
*dmrt1*	Doublesex- and mab-3-related transcription factor 1	Yes	Somatic	Up at day 7
*etnk1*	Ethanolamine kinase 1	Yes	Somatic Up stage 8	-
*-*	Homolog of Homo sapiens Transport-secretion protein 2.2	No	Somatic Up stage 8	-
*inha*	Inhibin	Yes	Somatic	Down at day 7
*mmp19*	Matrix metalloproteinase-19 precursor	Yes	A-spermatogonia	-
*plg*	Plasminogen	Yes	Somatic Up stage 8	Up at day 7
*cpvl*	Probable serine carboxypeptidase CPVL precursor	Yes	-	-
*-*	Putative uncharacterized protein	No	Somatic	-
*-*	Putative uncharacterized protein (Fragment)	-	Somatic	Up at day 7
*rbm47*	RNA-binding protein (FLJ20273), transcript variant 1	-	-	-
*slc9a3r1*	Solute carrier family 9 (Sodium/hydrogen exchanger), isoform 3 regulatory factor	Yes	-	-
*sptbn1*	Spectrin beta chain, brain 1	Yes	Somatic	-
*timp2*	Tissue inhibitor of metalloproteinase 2	Yes	Somatic Up stage 8	Up at day 7
*vt1*	Vasotocin-neurophysin	Yes	Somatic Up stage 8	Up at day 7

The corresponding genes segregated in Clusters 1or 2 and the response to Fsh was further identified as highly or moderately sensitive to trilostane in pairwise comparisons (Limma statistical test, p≤5%). When the information was available, we indicated the response to Lh (**^1^**) Sambroni et al., 2013), the testicular expression profile as well as the *in vivo* regulation by androgens ((^2^)Rolland et al., 2009 and (^3^) Rolland et al., 2013). -: not determined.

**Figure 5:**
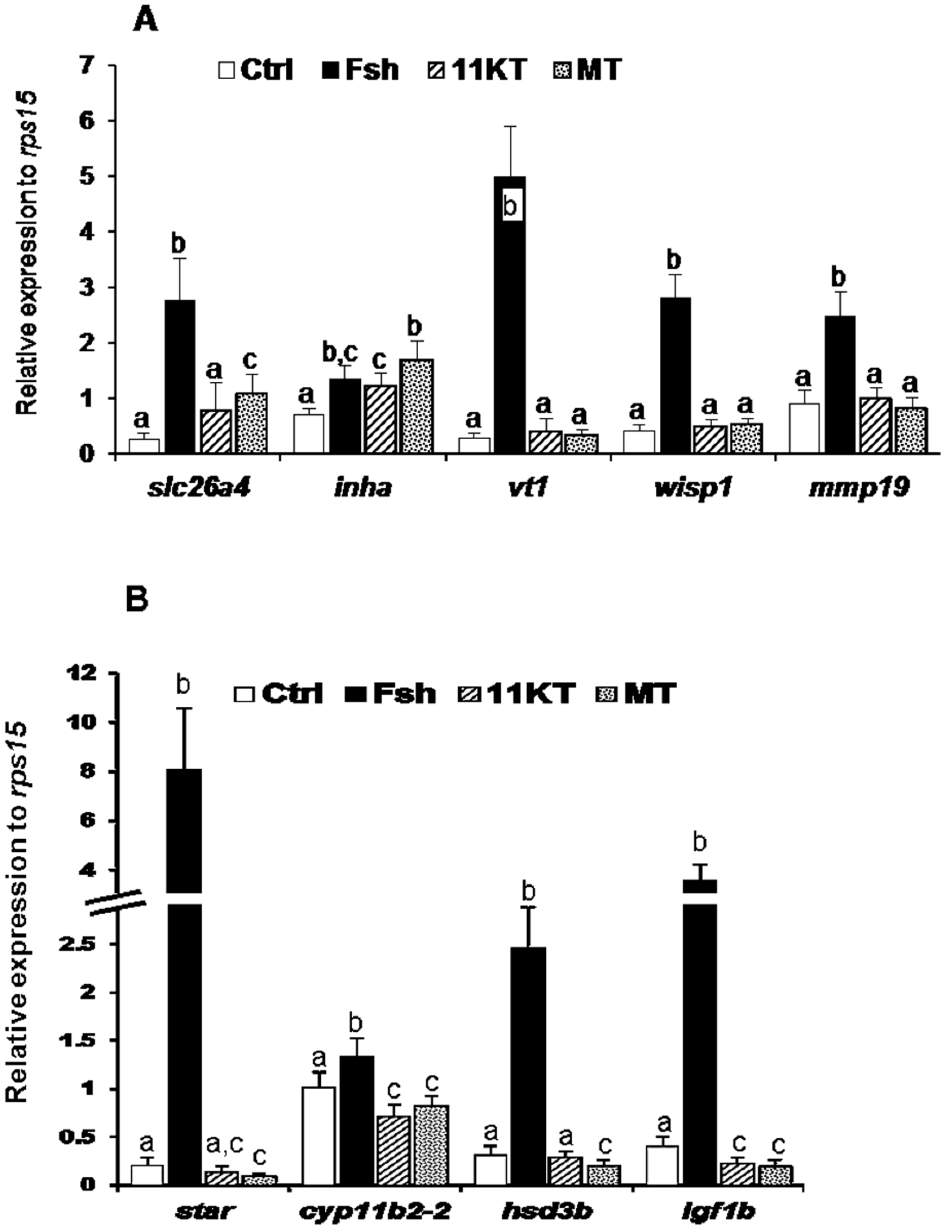
Real-time PCR measurement of candidate gene expression. Relative expression of selected mRNA transcripts in testicular explants cultured during 4 days in the absence (Ctrl) or presence of either Fsh (500 ng/mL), 11-ketotestosterone (11KT) or 17α-methyltestosterone (MT) at 300 ng/mL (∼10^−6^ M). Bars represent mean ± SD of 5 to 6 replicates. Expression data were normalized to the reference gene *rps15*. Different letters indicate that treatments are significantly different as determined by non-parametric Mann & Whitney test.

Additional evidence of steroid-dependent Fsh action was obtained in Experiment 2 where we analyzed the effects of 11KT and MT on the expression of candidate genes and compared in the same experimental design the effect of Fsh. As expected, Fsh and androgens did up-regulate the steady-state levels of two transcripts that were sensitive to trilostane, *slc26a4* and *inha* (Fig. 5A). However in our experimental conditions androgens were not able to stimulate *vt1*, *wisp1* and *mmp19*.

#### Fsh action independent of steroid production

Most interestingly Clusters 2 and 5 pointed out Fsh up-regulations or down-regulations, respectively, that were both maintained in presence of trilostane (Fig. 3). This demonstrates that Fsh could act on gene expression independently of the mediation of Δ4-steroids. Among the genes of interest in these groups (Table 3), we notice genes related to cholesterol biosynthesis (*hmgcr*, *abca1*), lipid metabolism (*fasn*) or steroidogenesis (*hsd3b1*). Other genes were encoding for neurohormones (*tac1*), growth factors (*mdka*, *amh*) and proteins involved in the cell cycle (*ccne1*, *ccng1*, *ing4* and *mcm7*) or in cell shape and cytoskeleton (*ezr*, *des*).

**Table 3:** Representative transcripts regulated by Fsh independently of Δ4-steroid production.

*Gene Symbol*	Gene description	Lh regulation^1^	Testicular expression profile^2^	*In vivo* androgen response^3^
**Genes up-regulated by Fsh**				
*hsd3b1*	*3-HSD 1 protein*	*Yes*	*Somatic Down stage 8*	*Down*
*hmgcr*	3-hydroxy-3-methylglutaryl-coenzyme A reductase	-	-	-
*aplp1*	Amyloid-like protein 1 precursor	-	-	-
*angptl7*	Angiopoietin-related protein 7 Precursor	-	Gonia A	Down
*abca1*	ATP-binding cassette sub-family A member 1	No	Somatic	-
*ctssb1*	Cathepsin S	No	Somatic Down stage 8	-
*ctss*	Cathepsin S precursor	No	Somatic	-
*slc7a3*	Cationic amino acid transporter 3 (CAT-3)	No	-	-
*ccng1*	Cyclin-G1	-	-	-
*eftud1*	Elongation factor Tu GTP-binding domain-containing protein 1			
*ezr*	Ezrin (p81) (Cytovillin) (Villin-2)	-	-	-
*fasn*	Fatty acid synthase	-	Somatic Up stage 8	-
*fra2*	Fos-related antigen 2	No	Gonia B	-
*hgfa*	Hepatocyte growth factor activator	-	-	Down
*hk1*	Hexokinase-1	-	-	-
*krt12*	Keratin 12	No	Somatic Down stage 8	-
*galns*	N-acetylgalactosamine-6-sulfatase precursor	Yes	-	Up at day 7
*odc1*	Ornithine decarboxylase	No	Somatic	-
*mdka*	Midkine-related growth factor (Pleiotrophin related)	No	Somatic	-
*tac1*	Protachykinin 1 precursor	No	Somatic Up stage 8	-
-	Putative uncharacterized protein	No	Somatic	-
*psmd1*	Proteasome (prosome, macropain) 26S subunit, non-ATPase, 1	No	-	Up at day 7
*rbm47*	RNA-binding protein (FLJ20273), transcript variant 1	-	-	-
*rhobtb3*	Rho-related BTB domain-containing protein 3	-	Somatic	-
*slc25a4*	Solute carrier family 25 member 43	Yes	Somatic	-
*tfpi2*	Tissue factor pathway inhibitor 2	No	Somatic Down stage 8	-
-	Toxin-1	Yes	-	-

Symbol	Gene Description	Lh negative response	Testicular expression profile	*In vivo* androgen response
**Genes down-regulated by Fsh**				
rpl30	*60S ribosomal protein L30*	*-*	*-*	*-*
amh	Anti-mullerian hormone	No	Somatic Down stage 8	Down
des	Desmin	No	-	-
mcm7	DNA replication licensing factor MCM7	Yes	-	-
ccne1	G1/S-specific cyclin-E1	Yes	Somatic Down stage 8	-
cenpa	Histone H3-like centromeric protein A	Yes	Meiotic/post meiotic	-
onmydab	Homolog of Homo sapiens HLA class II histocompatibility antigen, DQB1*0602 beta chain	Yes	Somatic	-
ing4	Inhibitor of growth family, member 4	No	Somatic Down stage 8	-
onmy-uba	MHC class I heavy chain	Yes	Somatic	-
nfkb1	Nuclear factor NF-kappa-B p105 subunit	Yes	-	-
pol	Pol polyprotein (Fragment)	-	-	-
akt3	RAC-gamma serine/threonine-protein kinase	-	-	-
fcf1	rRNA-processing protein FCF1 homolog	-	-	-
**Genes down-regulated by Fsh only in the presence of trilostane**				
*zfp658*	*BC043301 protein (Fragment)*	*-*	*-*	*-*
*purg*	Purine-rich element-binding protein gamma	-	-	-
*tmem106a*	Tmem106a protein (Fragment)	-	-	Up at day 7

Up-regulated genes mostly segregated in Cluster 2 whereas down-regulated genes segregated in Cluster 5. The response to Fsh was further identified as insensitive to trilostane in pairwise comparisons (Limma statistical test, p≤5%). When the information was available, we indicated the response to Lh (**^1^**) Sambroni et al., 2013), the testicular expression profile as well as the *in vivo* regulation by androgens ((**^2^**)Rolland et al., 2009 and (**^3^**) Rolland et al., 2013). -: not determined.

**Figure 6:**
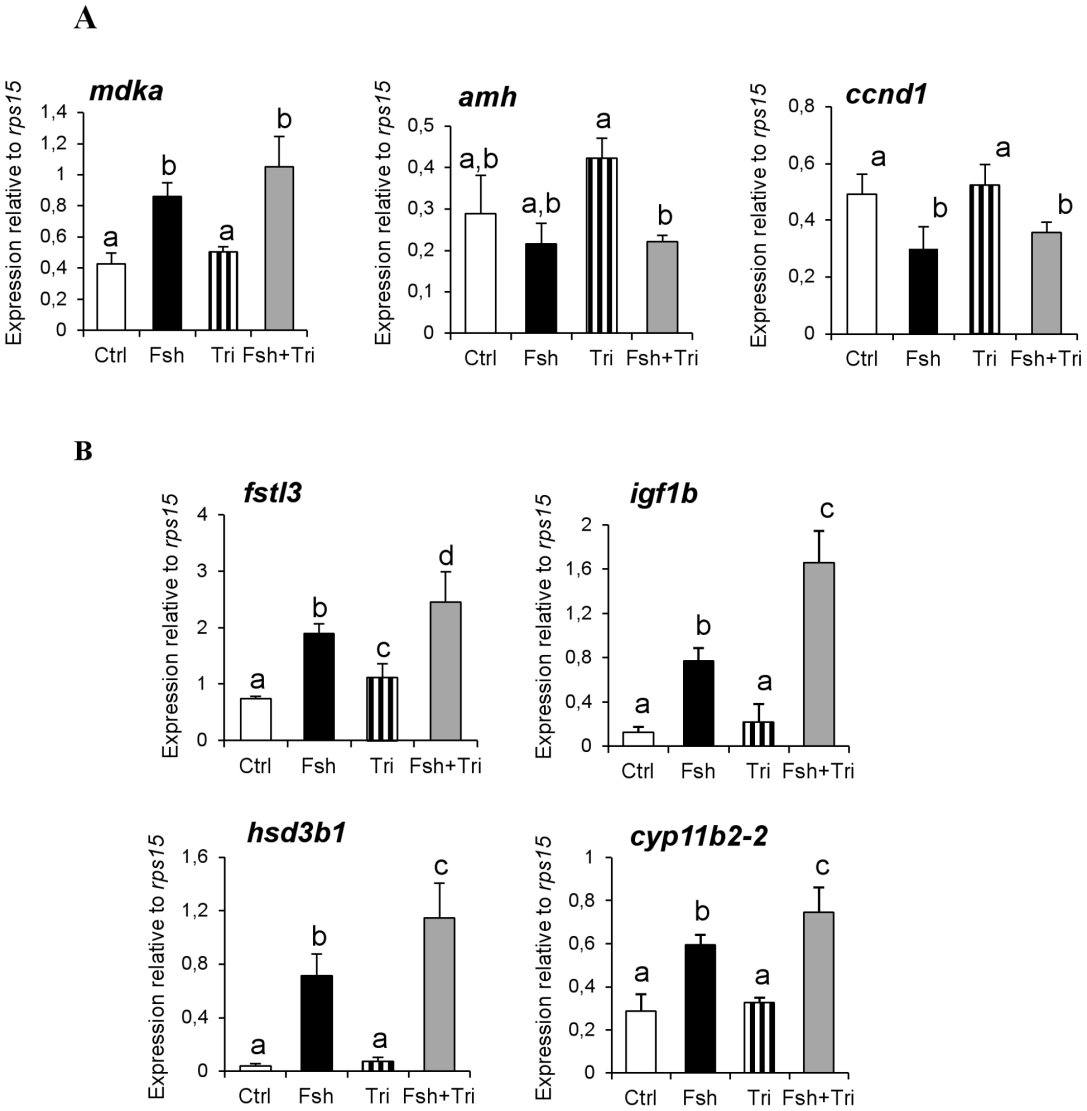
Steroid-independent action of Fsh on the steady-state level of mRNA transcripts measured by qPCR. The *mdka* and *hsd3b1* genes belong to Cluster 2 and the *amh* gene is segregated in Cluster 5. Four additional candidates previously demonstrated as being regulated by gonadotropins - *ccnd1*, *fstl3*, *igf1b/igf3* and *cyp11b2-2* - behave like genes of Cluster 2. Furthermore an increased response to Fsh in the presence of trilostane suggests an antagonism between Fsh and the Δ4- steroids. Bars represent mean ± SD of 5 to 6 replicates. Expression data were normalized to the reference gene *rps15*.

For selected genes of that category (Fig. 6), the qPCR measurement provided compelling evidence that Fsh action was independent of Δ4-steroid production. The regulation by Fsh either was not inhibited by trilostane (Fig. 6A: *mdka*, *amh* and *ccnd1*), or was even amplified in the presence of trilostane (see below and Fig. 6B).

#### Fsh and steroids show cooperative or antagonistic effects on gene expression

Cluster 3 regrouped genes for which basal expression was decreased by trilostane alone, suggesting a stimulatory action of Δ4-steroids, whereas the Fsh-stimulated expression level was not modified by trilostane. This indicates that Fsh and steroids regulate similarly those genes.

Cluster 4 was not expected because it grouped few genes for which i) Fsh alone had no significant modulatory effect, ii) trilostane stimulated basal gene expression, possibly revealing an inhibitory action of Δ4-steroids and iii) a stimulatory effect of Fsh was observed only in the presence of trilostane. This indicates that high levels of steroids induced by Fsh could prevent or mask Fsh action on gene expression. In coherence with the microarray data, qPCR measurements confirmed a group of transcripts characterized by an amplified response to Fsh in the presence of trilostane (Fig. 6B: *fstl3*, *hsd3b1*, *cyp11b2-2* and *igf1b*).

Direct evidence of the antagonistic effects between Fsh and steroids was demonstrated by comparing the impact of androgens and Fsh *in vitro* on candidate genes: while up-regulation by Fsh was confirmed, androgen treatment significantly down-regulated *igf1b*, *star*, *hsd3b1* and *cyp11b2-2* (Fig. 5B).

### Specific effects of Fsh are not mediated through Δ4-steroid

**Figure 7:**
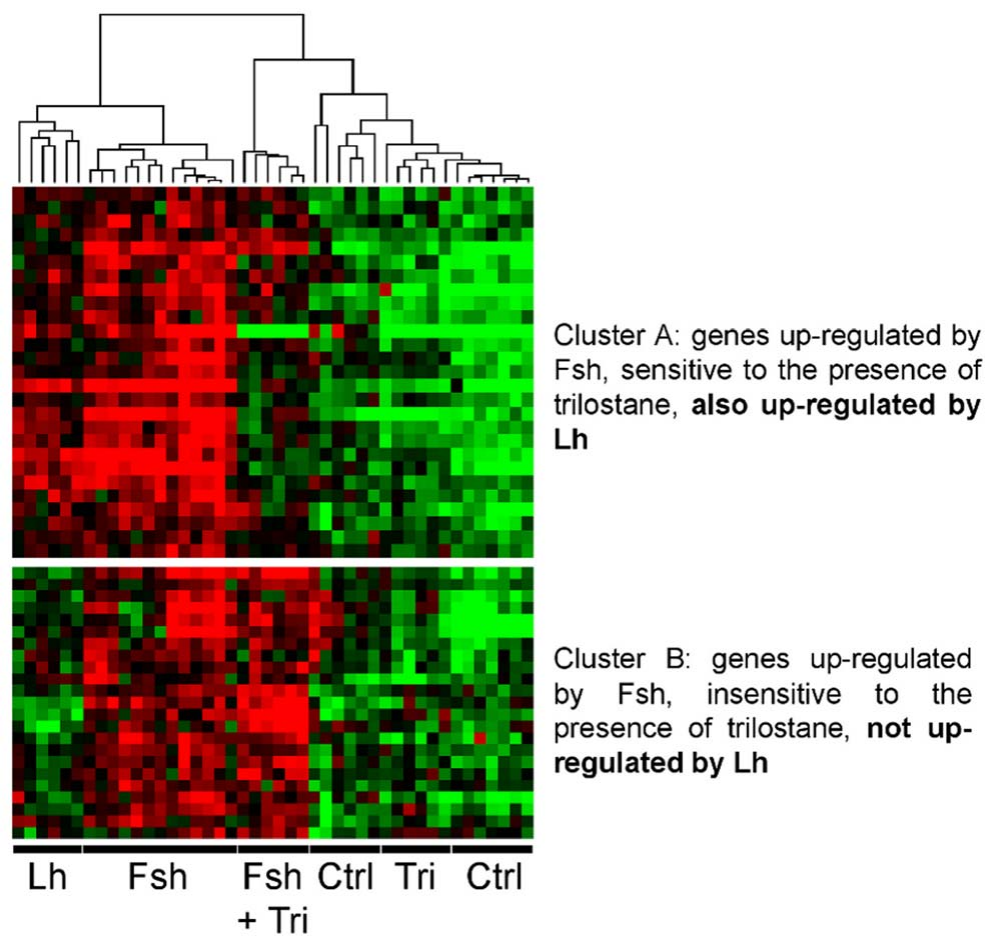
Meta-analysis: heatmap representation of the unsupervised hierarchical classification of the 58 clones found regulated by Fsh in the present study and in our previous study where Fsh and Lh effects on testicular transcriptome were measured (Sambroni et al., 2013).

In a previous study we showed that Fsh and Lh had both common and distinct effects on testicular transcriptome [10]. In the present study, we addressed the question of whether sex steroids mediated the common actions of Fsh and Lh, while the steroid-independent response to Fsh would correspond to genes specifically regulated by Fsh and not by Lh. To proceed to the meta-analysis, we combined data obtained in the present study with those reported previously [10] : after a step of normalization between the two experiments, we performed an unsupervised hierarchical classification including 44 arrays and the 58 clones found to be regulated by Fsh in the two studies (Fig. 7). Remarkably, we observed that the genes up-regulated by Fsh and sensitive to trilostane were also up-regulated by Lh (Cluster A). Conversely, the genes up-regulated by Fsh but insensitive to trilostane were not responsive to Lh (Cluster B).

## Discussion

Since both Fsh and Lh efficiently stimulate steroidogenesis in fish, deciphering their respective roles along the reproductive cycle remains a crucial question. Furthermore, whether Fsh acts directly on the spermatogenic compartment or through steroid production by Leydig cells remains unclear in many teleostean species studied so far. In this context, we addressed the question of whether the regulatory effects of Fsh on gene expression could be mediated independently of the production of biologically active steroids. Our conclusions are based on a large scale transcriptomic analysis.

To distinguish steroid-independent regulatory effects of Fsh from those mediated through the steroids, we used the trilostane, a known inhibitor of the 3β-hydroxysteroid dehydrogenase/D5-D4 isomerase (3β-HSD). Trilostane blocks the production of Δ4-steroids that include testosterone, 11 ketotestosterone, 17α,20β-dihydroxy-4-pregnen-3-one (DHP), and estradiol which are considered as the main biologically active sexual steroids in fish [29], [30]. As expected, we showed that the dose of trilostane used in the present study was effective in inhibiting the release of 11KT in the culture media, indicating that it inhibited the upstream processes in Δ4-steroid synthesis effectively. However, we cannot totally exclude the fact that the trilostane-insensitive actions of Fsh could in fact be mediated through the production of delta5 steroids, like dehydroepiandrosterone. Nevertheless, the delta 5 steroid pathway has been detected in immature trout ovaries [31], not in immature testis [32] and it is generally assumed that the biosynthesis of steroids in fish testis mainly follows the delta 4 pathway [33].

The analysis of the effects of trilostane on the changes induced by Fsh in the testicular transcriptome discloses two main mechanisms underlying the action of Fsh: the first mechanism involves the production of steroids, which in turn probably relay Fsh action. The second mechanism implies that Fsh acts independently of the Δ4-steroid mediation.

### Fsh action mediated by steroids

For specific transcripts mostly grouped in Cluster 1, the stimulatory effect of Fsh was totally or nearly suppressed when steroidogenesis was inhibited. This clearly indicates that Fsh acts indirectly on the corresponding genes through the production of sex steroids. This observation is in agreement with the steroidogenic activity of Fsh and the cellular expression of its cognate receptor on fish Leydig cells. Furthermore, several of these genes were also found to be up-regulated by androgen *in vivo* [34] or *in vitro* (this study). This supports the idea that androgens are the mediators of the effects of Fsh on those particular genes. Conversely, *in vitro* androgen treatment was unable to increase the steady-state level of mRNA of some genes (*vt1*, *wisp1* and *mmp19*) suggesting that the action of Fsh could be mediated by steroids other than 11KT and MT. Among the genes up-regulated through the mediation of steroids, we found the gonadotropin receptor transcripts, *fshr* and *lhcgr*. Although we cannot incriminate a particular steroid in the present study, our data are consistent with the androgen-induced increase of the steady-state levels of *fshr* and *lhcgr* in African catfish testis [35]. This observation combined with high gonadotropin plasma levels measured at the end of the reproductive cycle [20] could explain the large increase of *fshr* and *lhcgr* transcript expression observed in the spawning trout [17]. This finding highlights an efficient amplification loop of the gonadotropin signaling pathways at that stage.

The mediation through steroid production was also established for a few genes which were negatively regulated by Fsh. Among those, *inhba* encodes for the beta A subunit of activin. This subunit is part of growth factors involved in testis physiology and was previously found strongly down-regulated by androgens [34]. Another transcript in this group, *sl*, encodes for the somatolactin hormone, a fish hormone mainly expressed in the pituitary and capable to stimulate testicular androgen production in the gonads [36].

We noticed that in several cases the response to Fsh was significantly reduced but not fully suppressed in the presence of trilostane. Because in our experimental conditions the Fsh-induced steroid production was not totally suppressed by trilostane either (Fig. 1), we hypothesize that those genes are sensitive to low concentrations of steroids. However we cannot exclude a redundant regulation by Fsh and steroids for some of them, as evoked previously in mice for a few Sertoli cell transcripts [37].

Since in fish both Lh and Fsh induce steroid production, we hypothesized that the genes regulated by Fsh through steroid mediation (Cluster 1) should also respond to Lh *in vitro*. Recently, we demonstrated that Fsh and Lh have common but also distinct effects on gene expression in rainbow trout testis [10]. Our meta-analysis disclosed that a majority of Cluster 1 genes was similarly regulated by Fsh and by Lh in our previous study, supporting the idea that for those genes, Fsh acts similarly to Lh mainly through the mediation of steroids.

To highlight the biological significance of the hormonal regulations described above, we performed additional data mining to retrieve the expression profiles of the candidate genes during trout testis maturation which were previously reported [26]. We found that genes for which the action of Fsh was mediated by steroids displayed a large increase or a decrease at the end of the reproductive cycle, when both gonadotropin hormones and steroids exhibit high plasma levels in salmonids (data not shown). Such a convergence between high levels of circulating hormones and maximal or minimal gene expression levels during the reproductive cycle reinforces the physiological consistency of our data.

### Fsh action independent of steroids

Conversely, the effects of Fsh on genes grouped in Clusters 2 and 5 were maintained in the presence of trilostane, reflecting that a part of the Fsh action did not require the production of Δ4-steroid. Interestingly, in our meta-analysis, a majority of genes of this category (like *mdka*, *ctss*, *krt12* and *fosl2*) were also found preferentially up-regulated by Fsh when compared to Lh, reinforcing the idea of a steroid independent regulation. Several of these Fsh responsive genes including *amh*, *abca1*, *ezr*, *gapdhs*, *slc7a3*, *ccng1*, *cebpb*, and *fasn*, are known to be expressed in Sertoli cells in mice [38]. Furthermore, when comparing our data with those obtained in the rat after a 24 hour incubation of Sertoli cells with ovine FSH, we retrieve a number of genes (*ezr*, *hmgcr*, *odc1*, *fasn*, *des*) or pathways (Hgf system, amino acid transport, glycolysis) that are responsive to Fsh in the two studies [39]. The similarities between the regulations observed in trout and rat suggest that specific functions of Fsh would directly target Sertoli cells and have been, at least in part, conserved throughout the evolutionary process.

Our data indicate that steroid-independent effects of Fsh occurs also in Leydig cells. We noted that genes involved in steroidogenesis are induced by Fsh and not by steroids. These genes include *hsd3b1* and *star*. Our data and the expression of the Fshr previously reported in fish on Leydig cells [12]–[14], [16] suggest that Fsh could act directly on gene expression in those cells.

The *in vitro* Δ4-steroid independent action of Fsh on gene expression does not exclude the fact that steroids on their own could regulate the steady-state levels of some of these transcripts. In particular, genes encoding key factors involved in steroidogenesis were up-regulated by Fsh and down-regulated by androgens *in vivo*: *star*, *hsd3b1and cyp17a1* [34] or *in vitro*: *hsd3b1*, *star* and *cyp11b2-2* (this study). Such antagonistic regulatory effects suggest a short loop feedback by high concentrations of the sexual steroids that could be essential to allow a local fine-tuning of steroidogenesis. We also noted antagonistic effects between Fsh and steroids for genes involved in early germ cell proliferation and/or differentiation like *igf1b*.

### Fsh-regulated factors and testicular functions

We gave particular attention to several transcripts regulated by Fsh independently of the mediation of steroids, as they encode growth factor related products or cytokines, that may be key factors in the mechanism of Fsh action on germ cell proliferation/differentiation or on Leydig cell functions. In this category, we can cite anti-mullerian hormone (*amh*), insulin-like growth factor 1b (*igf1b*), hepatocyte growth factor activator (*hgfa*), follistatin-like 3(*fstl3*) and midkine (pleiotrophin related) (*mdka*). Except for *igf1b*, they are also found expressed in the mouse gonad [40]–[42].

Amh is known to prevent spermatogonial proliferation and differentiation in fish [43], [44]. Here, the down-regulation by Fsh of *amh* transcript was found to be insensitive to trilostane and therefore independent of Δ4-steroid production. This observation is consistent with the inability of androgens in regulating *in vitro amh* expression in trout (not shown) and in zebrafish [44] but in contradiction with 11KTdecreased amh mRNA levels in eel [43].

Igf1b (also named Igf3) is a new member of the Igf family which is preferentially expressed in teleost gonad [45]. Previous studies had demonstrated that Fsh and recombinant Igf1 stimulated spermatogonia proliferation in trout [21], [46]. More recently, we found that Fsh stimulated the expression of the *igf1b* gene (but not *igf1a* or *igf2*) suggesting that this Igf form is a major mediator relaying the Fsh action on trout spermatogonia proliferation [10].A similar conclusion was reached in zebrafish (Schulz and col., unpublished data). In the present study, high levels of androgens decreased the level of *igf1b* transcript. (Note that estrogens and the progestin DHP also down-regulate the gonadal *igf1b* mRNA level in zebrafish and tilapia [47]–[49]). The negative influence of androgens on the *igf1b* transcript in rainbow trout may therefore limit the accumulation of spermatogonia during the mid and late testicular stages. In addition, we previously showed that administration of androgens tended to stimulate the expression of germ cell genes involved in meiotic differentiation [34]. Altogether, our data suggest a sequential cooperation between Fsh and sex steroids in trout: a primary function of Fsh in prepubertal males would be to stimulate the active accumulation of spermatogonia; in animals undergoing pubertal maturation, the increasing production of steroids would limit the proliferation of spermatogonia and favor their differentiation.

Hgf, which is activated upon endoproteolysis by Hgfa, was shown to block apoptosis and stimulate the proliferation of germ cells in the prepubertal rat testis [50]. Fstl-3 is a glycoprotein that binds and inhibits the action of TGFβ ligands such as activins, which have numerous developmental and regulatory activities within the gonads. The role of midkine/pleiotrophin family, secreted heparin-binding cytokines, in the regulation of testicular function, is unknown in fish and poorly documented in vertebrates. However an interesting study reported an increase of germ cell apoptosis in mice having a dominant negative mutation of pleiotrophin [51]. Midkine increases activity of mitotic pathways in primordial germ cells in vitro, keeping them in a proliferative, less differentiated state [41]. Considering their functions, these 3 factors point to new strong candidate pathways that could specifically mediate Fsh regulation of germ cell survival or proliferation in fish.

**Figure 8:**
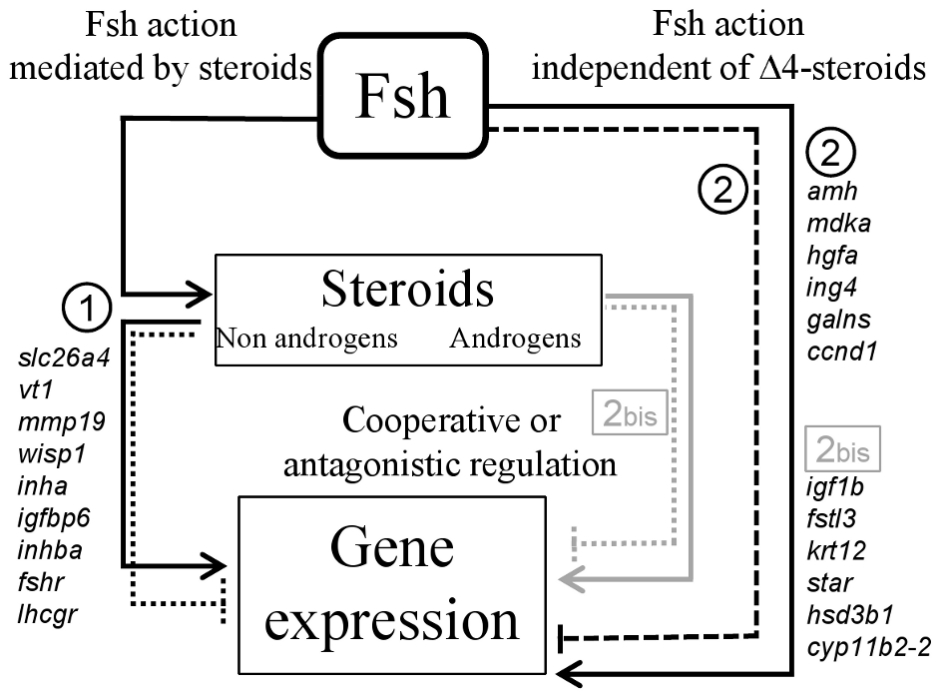
Summary of the mechanisms underlying Fsh action on gene expression in rainbow trout testis. 1- The primary action of Fsh is to stimulate steroidogenic cells to produce steroids which in turn regulate gene expression. 2- Fsh exerts specific regulatory effects independently of steroid mediation. 2 bis- In some cases steroids could have either an antagonistic or a redundant effect on gene expression. Plain lines with an arrow head indicate stimulatory effects whereas dotted lines illustrate inhibitory effects.

In summary (Fig. 8), this study provides the first large scale evidence that Fsh controls gene expression in fish testis through two different mechanisms: the first one requires the production of steroids whereas the second mechanism requires a steroid-independent pathway. We point out new candidate pathways that could be involved in the primary effects of Fsh on early spermatogonial and/or Sertoli cell proliferation in trout, then on meiosis initiation, when Lh is not yet secreted. Finally, a few cooperative or antagonistic effects between Fsh and sex steroids are demonstrated. We anticipate that the knowledge gained from this study will provide new insights on the specific role of Fsh and on its cooperation with steroids in regulating fish spermatogenesis.

## Supporting Information

Table S1 **Sequences of primers used in qPCR experiments.** The gene symbol, the accession number and the sequence of forward and reverse primers (5′-3′) used for qPCR measurements are indicated.
(DOCX)

File S1 **Steroid-mediated and steroid–independent actions of Fsh on gene expression.** The searchable excel file summarizes the effect of trilostane on the Fsh responsiveness of 102 testicular genes found to be regulated using the microarray approach (Clusters 1 to 5) and provides a comprehensive annotation including “Clone Name”, “Gene Symbol”, “Gene name”, GeneOntology terms and IDs (“Biological process”, “Molecular function” and “Cellular component”). Additional information, extracted from previous studies, is also reported and includes Lh responsiveness at stage I–II and stage III (Sambroni et al., 2013), testicular expression profile (Rolland et al., 2009) and androgen responsiveness (Rolland et al., 2013). The file also provides the quantile-quantile normalized expression data (Log-2 transformed) of the 102 clones found to be significantly regulated.
(XLSX)
