# Supplementary material for: Fsh Controls Gene Expression in Fish both Independently of and through Steroid Mediation
Source: PLoS One. 2013 Oct 23;8(10):e76684. doi: 10.1371/journal.pone.0076684 (PMC3806798; doi:10.1371/journal.pone.0076684)
Supplement: Table S1 — Sequences of primers used in qPCR experiments. The gene symbol, the accession number and the sequence of forward and reverse primers (5′-3′) used for qPCR measurements are indicated. (DOCX) [file pone.0076684.s001.docx]

**Table S1:** Sequences of primers used for q-PCR experiments.

| **Gene symbol** | **Acc number** | **Forward primer** | **Reverse primer** |
| --- | --- | --- | --- |
| *rps15 (ref)* | [ACO08621](http://www.ncbi.nlm.nih.gov/protein/ACO08621) | CCTGGGGGAGTTCTCTATCACCT | GGGATGAAACGGGAAGAATGTGT |
| *fshr* | [AF439405](http://www.ncbi.nlm.nih.gov/nuccore/AF439405) | TCAGTCACCTGACGATCTGCAA | TCCTGCAGGTCCAGCAGAAACG |
| *lhcgr* | [AF439404](http://www.ncbi.nlm.nih.gov/nuccore/AF439404) | CTTCTCAACCTCAATGAAATCTTC | GGATATACTCAGATAACGCAGCTT |
| *igf1b* | [CX025953](http://www.ncbi.nlm.nih.gov/nucest/CX025953) | GTGTGGAGACCGTGGATTTT | CACAATTCCCTTCCCTCTCA |
| *igfbp6* | [DQ190459](http://www.ncbi.nlm.nih.gov/nucleotide/80972566) | GCTCAATAGTGTTCTGCGTGG | CTTGGAGGAACGACACTGCTT |
| *star* | [AB047032](http://www.ncbi.nlm.nih.gov/nuccore/AB047032) | GAGTTGTTAGGGCAGAGAAC | CAACCCTTTAAATCTATGCTTA |
| *hsd3b1* | [S72665](http://www.ncbi.nlm.nih.gov/nuccore/S72665) | TACAGTGCCTGGAAGAGATCAGA | ACCCTGTGAAGCTCACTGTATAA |
| *cyp11b2-2* | [AF217273](http://www.ncbi.nlm.nih.gov/nuccore/AF217273) | CTGGGACATGTGTCCAGGCA | CTGGATCCTGAAACACGTCA |
| *fstl3* | [NM_001160487](http://www.ncbi.nlm.nih.gov/nuccore/238231552) | ACCGCTGAAGTCCGAGTTGC | GCAGGTGGCTCTGTGGAGGT |
| *amh* | [Q5XZF0](http://www.ncbi.nlm.nih.gov/protein/Q5XZF0) | GGGAATAACCATGCTATCCTGCTTAA | CTCCACCACCTTGAGGTCCTCATAGT |
| *inha* | [Q9DED3](http://www.ncbi.nlm.nih.gov/protein/Q9DED3) | CCAGCTCTGACTCTACCTGTGAT | CCTGGTTGTCGAGGGAGGATTG |
| *inhba* | [D88463](http://www.ncbi.nlm.nih.gov/nuccore/D88463) | AGGGCAAGGTGAACATACAG | CCTCGTGTCCACCATCTTCTC |
| *inhbb* | [AB044566](http://www.ncbi.nlm.nih.gov/nuccore/AB044566) | GTTTCGCAGAGACAGATGAG | GTCACATACAGGTGCTGGTT |
| *slc26a4* | [NM_001165915](http://www.google.fr/url?sa=t&rct=j&q=NM_001165915.1&source=web&cd=1&cad=rja&ved=0CC8QFjAA&url=http%3A%2F%2Fwww.ncbi.nlm.nih.gov%2Fnuccore%2FNM_001165915.1&ei=RY0TUtuvA4H40gW1uoHICw&usg=AFQjCNF7jkd5LmjA34lqqOBmT8p3g8xJxA) | CGGCACAAACATATACAGGAA | CCACCGTGACTCTCAATCGTTCT |
| *vt1* | [CA375992](http://www.ncbi.nlm.nih.gov/nucest/CA375992) | GAGGCTGGAGGAAGAGTGTG | TTCTGTTTGCTGGGTGACTG |
| *mmp19* | [BX081049](http://www.ncbi.nlm.nih.gov/nucest/BX081049) | AGTTCTGAGGGAGTGTGTGG | TGTTGTGAGGGATAGGAAGG |
| *mdka* | [AF149802](http://www.ncbi.nlm.nih.gov/nuccore/AF149802) | CAGTGTTCTTGGTCTGCCTAA | CTTGGTCTTGACTCCAGTTGA |
| *wisp1* | [BX868220](http://www.ncbi.nlm.nih.gov/nucest/BX868220) | TTCAACTCAACAGGAAGAGC | AACTCAAGGAGGGTCAAGAT |
| *ccnd1* | [CA374091](http://www.ncbi.nlm.nih.gov/nucest/CA374091) | GTCCCTTTAACTGCAGAGAAGT | ATCGTGAGGTGTTACTGATGCT |
